# High Commitment Work System and Employee Proactive Behavior: The Mediating Roles of Self-Efficiency and Career Development Prospect

**DOI:** 10.3389/fpsyg.2022.802546

**Published:** 2022-04-05

**Authors:** Yang Shi, Man Cao

**Affiliations:** ^1^Business School, Changshu Institute of Technology, Suzhou, China; ^2^School of Economic and Management, Southeast University, Nanjing, China

**Keywords:** high commitment work systems, employee proactive behavior, self-efficiency, career development prospect, conformity value

## Abstract

At present, proactive behavior has become a major concern in the field of organizational behavior. Drawing from the proactive motivation theory, this article proposes the influence of a high commitment work system (HCWS) on employees’ proactive behavior and constructs the mediation model, including self-efficacy and career development prospect. Moreover, conformity values as a micro context factor are used to illustrate the process that affects employees’ proactive behavior. Analyzing the matched data from 117 enterprises and 1,055 employees, this article finds that HCWS are positively related to employees’ proactive behavior. This article also finds that self-efficacy and career development prospect are mediated by the relationship between HCWS and employees’ proactive behavior. Conformity value moderates the positive relationship between self-efficacy and employees’ proactive behavior, but it does not moderate the positive relationship between career development prospect and employees’ proactive behavior. This study sheds light on whether and how line managers’ leadership influences the human resource management (HRM) process.

## Introduction

In the face of the increasing complexity of the global environment, organizations increasingly need to rely on the proactive behavior of employees to solve the problems at any time (Campbell, 2000; Wu et al., 2018). Previous studies have also shown that proactive behavior can effectively improve employee performance (Griffin et al., 2007), promote employee innovation (Herrmann and Felfe, 2014), and bring beneficial results for the team and organization, such as improving team learning (Druskat and Kayes, 2000) and team performance (Kirkman and Rosen, 1999). Therefore, how to deeply stimulate the initiative of employees, and improve the team and organizational effectiveness, has a very important practical significance for the sustainable development of the organization.

Proactive behavior refers to the behavior that individuals try to change themselves or their environment. Since the 1990s, scholars have discussed the influence of different types of factors on proactive behavior, such as individual personality (Bateman and Crant, 1993), work characteristics (Parker et al., 2006), and organizational context factors (Griffin et al., 2007). However, most of the existing studies tend to focus on one of these factors, which make the current research on proactive behavior in a fragmented state (Hong et al., 2016). In this regard, it is necessary to combine the organizational situational factors and individual factors and, comprehensively, considers the complex impact of the combination on proactive behavior, which can further clarify the formation process of proactive behavior (Parker et al., 2010).

High commitment work system (HCWS), as one of the important contents of strategic human resource management (HRM), is to enhance the organizational commitment of employees through a series of interrelated and collaborative HRM measures (Wood and Albanese, 1995; Xiao and Björkman, 2006; Boon and Kalshoven, 2014; Fleming and Spicer, 2016). In the field of strategic HRM, scholars have defined the HRM system according to different research perspectives, such as high performance work system (HPWS), HCWS, and high involvement work system (HIWS). Compared with HPWS and HIWS, HCWS is different from other HRM systems in terms of connotation and measurement, and is characterized by strengthening staff skills, motivation, and information sharing. HCWS focuses on the development of long-term relationship between organization and employees (Tsui et al., 1997; Xiao and Björkman, 2006; Kwon et al., 2010) and strives to build the psychological connection between individual goals and organizational goals, which will shape employees’ attitude and behavior (Boselie et al., 2005; Xiao and Björkman, 2006; Iverson and Zatzick, 2007; Chen, 2018). At the same time, HCWS emphasizes and allows employees to complete work-related tasks consistent with organizational goals (Arthur, 1994; Chiang et al., 2011; McClean and Collins, 2011), which undoubtedly has a strong incentive effect on employees to implement proactive behavior. Some researchers have explored how HPWS interact with leader-member exchange (LMX) to predict employees’ proactive behavior (Lin et al., 2021). However, only few studies reveal the relationship and mechanism between HCWS and employee proactive behavior.

At the same time, throughout the previous research on proactive behavior, scholars tend to focus on general cognitive motivation, such as intrinsic motivation (Parker et al., 2006; Hong et al., 2016), and change responsibility sense (Lebel and Patil, 2018). But some studies lack of identification of specific cognitive motivation from the previous dependent variables, which leads to the existing research on cognitive motivation of proactive behavior, is relatively few (Allen et al., 2003; Meyer and Allen, 1984). Therefore, this study focuses on the typical cognitive motivation, such as self-efficacy, and identifies career development prospect stimulated by HCWS in order to enrich and expand the understanding of the formation process of proactive behavior. On the one hand, HCWS devotes to the long-term development of employees through a variety of measures and directly opens up an upward career channel for capable employees through internal promotion and other measures (Collins and Smith, 2006). On the other hand, it provides more challenging work experience for employees through giving responsibility and authorization, which will directly or indirectly make employees feel higher career development prospect. When employees can perceive the improvement of future career development prospect in their work, they are more willing to pursue their own goals and take proactive behavior.

In addition, based on the proactive motivation theory, in addition to the role of organizational context factors, individual factors also have a significant impact on proactive behavior (Bindl and Parker, 2011). Individual evaluation of “whether it is worth doing” can promote or inhibit the formation of proactive behavior (Parker et al., 2010). Therefore, this study focuses on the impact of personal values. Personal values are the guiding principles of individual life (Schwartz, 1992) and constitute an important basis for individuals to evaluate whether proactive behavior is worth doing. Therefore, when HCWS provides individuals with a variety of positive psychological states, different individual values affect the whole motivation process.

**FIGURE 1:**
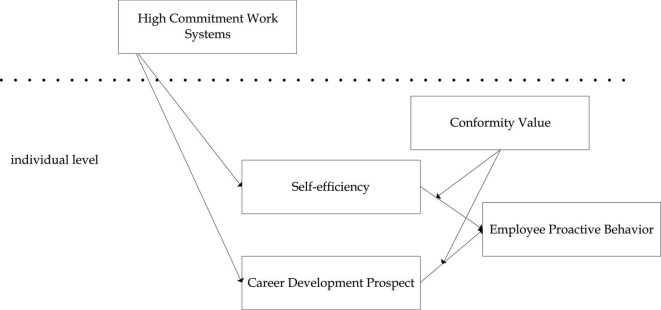
Research framework.

To sum up, this article deeply discusses the influence mechanism of HCWS on employee proactive behavior and constructs a dual mediation model, including self-efficacy and career development prospect. This article also examines the moderating effect of conformity values on the process of employee proactive behavior. This article systematically investigates the influence of organizational situational factors (e.g., HCWS) and individual factors (e.g., conformity values) on employee proactive behavior, which will help deepen the understanding of the process of proactive behavior (Figure 1).

## Theory Background and Hypotheses

### High Commitment Work System and Proactive Behavior

The HCWS refers to a series of collaborative HRM measures to achieve a performance improvement by stimulating employees’ commitment (Wood, 1996; Xiao and Tsui, 2007; Kwon et al., 2010; Kim and Wright, 2011; Ceylan, 2013). These measures include providing internal promotion opportunities, emphasizing individual potential selection, team performance-based compensation, participatory decision-making, and extensive training (Collins and Smith, 2006). Generally speaking, HRM practice can be divided into control type and commitment type (Arthur, 1994; Hauff et al., 2014). The goal of the controlled HRM practice is to reduce labor costs or improve efficiency by making employees obey specific rules and procedures. In contrast, the committed HRM practices emphasize the construction of a psychological connection between the organization and employees, so that employees can identify the organizational goals, and provide autonomy for employees to better complete the work-related tasks (Whitener, 2001; Kim and Wright, 2011). Therefore, the HCWS creates a good environment for employees to perceive that the organization attaches importance to its interests, and also makes employees more willing to commit and strive to achieve organizational goals. At the same time, a large number of studies also show that HCWS can bring many positive results, such as improving employee work engagement (Boon and Kalshoven, 2014; Chen, 2018), promoting employee work commitment (Whitener, 2001), stimulating employee creativity (Ceylan, 2013; Chang et al., 2014), and ultimately effectively improving organizational effectiveness (Hauff et al., 2014).

The HCWS also provides appropriate facilities for the motivation of employees’ proactive behavior. First of all, the selection process in the HCWS can help the organization select employees with knowledge and potential, and help employees acquire knowledge about the work-related tasks and improve relevant skills needed in work (Wood and Menezes, 1998; Hauff et al., 2014). These necessary knowledge and skills enable employees to have the hard conditions to carry out proactive behavior (Parker et al., 2006; Farndale et al., 2011). Second, HCWS can provide employees to participate in decision-making and enhance employees’ work autonomy, so that employees have more opportunities to improve work proactively (Kim and Wright, 2011). Third, internal promotion and other measures in the HCWS stimulate employees’ work motivation, which makes employees more willing to participate in the work process and actively pursue their career goals (Chiang et al., 2014; Chen, 2018). Finally, HCWS reflects the idea of long-term investment for employees, which constructs the psychological connection between the organization and employees and makes employees willing to put extra efforts for organizational goals and personal goals (Xiao and Tsui, 2007). Therefore, this study proposes the following hypothesis:

***Hypothesis 1:*** *HCWS has a positive impact on employee proactive behavior.*

### Mediating Role of Self-Efficacy

According to the proactive motivation theory, personal experience is driven by two kinds of cognitive motivations, namely, “can do” and “have reason to do.” The previous expectation valence theory also provides an additional explanation for the cognitive motivation of proactive behavior. For the cognitive motivation of “can do,” this study focuses on the variable of self-efficacy. Bandura (1977) put forward the concept of “self-efficacy” for the first time and defined self-efficacy as the evaluation of an individual ability to perform the task. Self-efficacy is not immutable and is influenced by the information received by individuals. It is generally believed that self-efficacy is a positive factor in HRM. Employees with a high sense of self-efficacy are more likely to adjust their emotions, actively meet the challenges, and improve the effectiveness of HRM. As one of the important messages of HRM, HCWS can have a positive impact on employees’ self-efficacy (Hauff et al., 2014), which will make employees more confident to complete their work and improve their self-confidence in work (Xiao and Björkman, 2006).

Self-efficacy also plays an important role in the initiation and maintenance of proactive behavior. When individuals have a higher sense of self-efficacy, they will be more confident to implement proactive behavior (Sonnentag and Grant, 2012). Even if there are various difficulties and obstacles in the process of implementation, individuals will try their best to take action to achieve the goal. By comparison, when individuals have a lower sense of self-efficacy, they will prefer to take the evasive way (Zhao et al., 2005). From this point of view, self-efficacy can have a positive impact on proactive behavior. The research results of Campbell (2000) also show that employees with a high level of self-efficacy are more likely to make proactive behavior. To sum up, HCWS can improve employees’ self-efficacy and promote the generation of employees’ proactive behavior. Therefore, this study proposes the following hypothesis:

***Hypothesis 2:*** *self-efficacy plays a mediating role between HCWS and proactive behavior.*

### Mediating Role of Career Development Prospect

Career development prospect means that employee will be given increased responsibilities, challenging tasks, and learning opportunities to enhance the possibility of career development in the organization. Previous studies have found that employees can perceive career development prospect not only through the promotion of their level in the organization but also through the increased responsibilities and challenges in their current jobs. HCWS emphasizes the connection between organizational goals and individual goals, which will undoubtedly have a positive impact on the career development prospect of employees. First of all, the provision of career development prospect, such as internal promotion, can make employees realize that the organization gives great importance to the development of employees (Collins and Smith, 2006) and directly know the possibility of promotion in the organization. Second, extensive training can help employees improve their work-related knowledge and skills, which undoubtedly provides the necessary ability reserve for the career development of employees. Finally, organizational measures, such as allowing employees to participate in decision-making, give employees more responsibilities for the perspective of work. In such a situation, HCWS can increase employees’ work responsibility and challenging experience and also promote employees’ perception of career development prospect.

In addition, career development prospect is also an important factor affecting work behavior. Previous empirical studies have shown that career development prospect can promote the improvement of employees’ work efficiency and make employees more willing to work in the company (Okurame, 2012). At the same time, when employees perceive that their career development prospect in the organization is very high, they will be more willing to meet challenges and overcome obstacles in the process of organizational development and show more proactive behavior. To sum up, HCWS can enhance employees’ career development prospect perception, which further stimulates employees’ proactive behavior. Therefore, this study proposes the following hypothesis:

***Hypothesis 3:*** *career development prospect plays a mediating role between HCWS and proactive behavior.*

### Moderating Role of Conformity Value

Personal values can guide individuals’ behavioral choice and evaluation and help individuals evaluate whether a certain behavior is “worth doing” (Schwartz, 1994). The person who values conformity seeks obedience to clear rules and structures. They gain a sense of control through doing what they are told and conforming to the agreed laws and statutes (Schwartz, 1992).

Due to the spontaneity and transformative behavior, the process of implementing proactive behavior is full of fuzziness and danger (Glaser et al., 2015). For example, when individual dominant values tend to strictly comply with other people or social expectations, the individual thinks that proactive behavior may involve other people’s interests and is not worth taking risks to do it. In this situation, even if the individuals in the organization have a strong sense of self-efficacy, they will implement less proactive behavior to meet the social expectations. On the contrary, when the individual conformity values are weak, they will be less affected by the expectation of the mainstream society. At this time, employees with high self-efficacy are more free to play their own enthusiasm and proactive and produce more proactive behavior. Therefore, conformity value plays a negative moderating role between self-efficacy and proactive behavior. When the conformity values are high, the positive relationship between self-efficacy and proactive behavior is weaker. When the conformity values are low, the positive relationship between self-efficacy and proactive behavior is stronger.

Similarly, when conformity values are high, even if employees perceive a high prospect of career development, they have the motivation to take the proactive and are willing to work hard to achieve their career goals. On the contrary, when conformity values are low, employees will work more proactively to achieve their own goals. That is to say, conformity value plays a negative moderating role between career development prospect and proactive behavior. When the conformity values are high, the positive relationship between career development prospect and proactive behavior is weaker. When the conformity values are low, the positive relationship between career development prospect and proactive behavior is stronger. Therefore, this study proposes the following hypotheses:

***Hypothesis 4:*** *Conformity values negatively moderate the relationship between self-efficacy and proactive behavior.*
***Hypothesis 5:*** *Conformity values negatively moderate the relationship between career development prospect and proactive behavior.*

## Materials and Methods

### Sample and Procedure

We collected data from 150 enterprises in Jiangsu, Anhui, Guangdong, Sichuan, Shandong, and other places in China. To ensure the rigor of research design and reduce the influence of homologous method deviation, we used two sources of human resources managers and employees and carried out the paired collection. Among them, human resource department managers evaluated the HCWS, and employees evaluated the self-efficacy, career development prospect, conformity values, and proactive behavior. The whole data collection process is completed with the assistance of enterprise executives. Before the formal investigation, the executives of each enterprise are contacted to explain the purpose, process, and precautions of the investigation in detail. Then, the specific survey time was agreed, and the formal survey questionnaire was mailed to the person in charge of each enterprise. Finally, the executives of each enterprise distributed it on behalf of the investigator and then mailed it to the investigator after recycling.

In this study, a total of 150 questionnaires were distributed to human resources department managers and 1,500 employees. Finally, 146 questionnaires were collected from managers and 1,287 employees. The recovery rates were 97.3 and 85.8%, respectively. After eliminating too many missing and highly consistent questionnaires, 117 valid questionnaires were obtained for managers and 1,055 employees, with effective recovery rates of 78 and 70.3%, respectively. In terms of sample structure, 54.5% were men and 45.5% were women. Employees under 25 years accounted for 17.4%, employees aged 26–30 accounted for 33.4%, employees aged 31–35 accounted for 25.9%, employees aged 36–40 accounted for 10.5%, and employees aged 41 and above accounted for 12.8%. In terms of educational level, 11.5% were below senior high school, 30.9% were junior college, 52.5% were undergraduates, and 5.1% were master’s degree or above. In terms of industries, 61.5% of enterprises are manufacturing industries and 38.5% are non-manufacturing industries. In terms of enterprise scale, enterprises with less than 100 employees accounted for 16.2%, enterprises with 101–500 employees accounted for 37.6%, enterprises with 501–1,000 employees accounted for 18.0%, and enterprises with 1,001 employees or more accounted for 28.2%.

### Measures

All scales were scored by the 7-point Likert-type method from 1—completely disagree to 7—completely agree.

1. HCWS: The scale was developed by Xiao and Björkman (2006), including 15 items. The typical topics are “internal selection, not external recruitment,” “performance appraisal emphasizes future skills development, not individual performance,” and “emphasize team work, collectivism, not individual struggle.” The Cronbach’s α coefficient of the scale is 0.914.
2. Career development prospect: The scale was developed by Reiche et al. (2011), including 5 items. The typical topics are “the knowledge, skills and contacts I have gained/will gain in the course of my tasks increase my chances of staying in this company in the future.” The Cronbach’s α coefficient of the scale is 0.910.
3. Self-efficacy: The scale was developed by Schwarzer and Born (1997), including 10 items. The typical topics are “if I try my best, I can always solve the problem.” The Cronbach’s α coefficient of the scale is 0.905.
4. Conformity values: The scale was developed by Schwartz (1992), including 4 items. The typical topics are “I think people should know how to obey orders” and “In my opinion, we should abide by the rules in any case, even if no one around us pays attention.” The Cronbach’s α coefficient of the scale is 0.852.
5. Proactive behavior: The scale was developed by Frese et al. (1997), which includes 7 items. The typical topics are “I solve the problem actively” and “I will take the proactive immediately, even when others do not do it.” The Cronbach’s α coefficient of the scale is 0.910.
6. Control variables: According to the previous literature (Wu and Parker, 2017), this study controlled the gender, age, and education level of the individual level. At the organizational level, this study controls the industry, enterprise size, and enterprise years.

## Results

### Descriptive Statistics

In Table 1, the means, standard deviations, and correlations of all studied variables are presented.

**TABLE 1:** Means, standard deviations, and correlations among study variables.

Variable	Mean	SD	1	2	3	4	5	6
**Organizational variables**								
1. Industry	0.62	0.49						
2. Enterprise scale	3.68	1.43	0.239**					
3. Enterprise years	18.15	15.50	0.150	0.327**				
4. High commitment work system	5.00	0.99	0.066	–0.004	0.161			
**Individual level variables**								
1. Gender	1.46	0.50						
2. Age	3.76	1.43	−0.118**					
3. Education level	2.94	1.17	–0.015	−0.210**				
4. Self-efficacy	5.31	0.88	–0.051	–0.001	–0.021			
5. Career development prospect	4.86	1.15	−0.090**	–0.013	–0.014	0.478**		
6. Conformity values	6.08	0.87	0.062*	0.027	−0.098**	0.456**	0.286**	
7. Proactive behavior	5.57	0.90	–0.011	–0.014	–0.029	0.636**	0.465**	0.495**

Level 1, n = 1,055; level 2, n = 117. *p < 0.05, *p < 0.01, *p < 0.001.

Industry (1 represents manufacturing enterprises and 0 represents non-manufacturing enterprises); enterprise scale (1 represents less than 50 people, 2 represents 50–100 people, 3 represents 101–500 people, 4 represents 501–1,000 people, 5 represents 1,001–2,000 people, and 6 represents more than 2,001 people); gender (1 for men and 2 for women); age (1 for 25 years old or below, 2 for 26–30 years old, 3 for 31–35 years old, 4 for 36–40 years old, 5 for 41–45 years old, 6 for 46–50 years old, 7 for 51–55 years old, and 8 for 55 years old or above); education level (1 for senior high school and below, 2 for junior college, 3 for bachelor’s degree, and 4 for master’s degree and above).

### Confirmatory Factor Analyses

We used LISERL6 software to conduct confirmatory factor analysis on proactive behavior, career development prospect, self-efficacy, and conformity values. As shown in Table 2, the four-factor model has a high degree of fitting (χ^2^ = 330.73, *df* = 48, RMSEA = 0.076, CFI = 0.970, TLI = 0.958).

**TABLE 2:** Confirmatory factor analysis of discriminant validity.

Model	Variables contained	X^2^	df	RMSEA	CFI	TLI
Basic model	PCP, GSE, CON, PB	330.73	48	0.076	0.970	0.958
Model 1	PCP + GSE, CON, PB	1,977.54	51	0.192	0.793	0.732
Model 2	PCP, GSE + CON, PB	1,260.23	51	0.152	0.870	0.832
Model 3	PCP, GSE, CON + PB	1,384.12	51	0.159	0.857	0.814
Model 4	PCP, GSE + CON + PB	2,149.79	53	0.196	0.774	0.719
Model 5	PCP + GSE + PB, CON	2,888.26	53	0.228	0.695	0.620
Model 6	PCP + GSE + PB + CON	3,828.910	54	0.261	0.594	0.503

*PCP represents career development prospect, GSE represents self-efficacy, CON represents conformity values, and PB represents proactive behavior.*

### Hypothesis Testing

In this study, given the nested nature of the data, we used HLM6 to estimate the proposed model. First, the zero model was used to test the proactive behavior, self-efficacy, and career development prospect, in order to examine the necessity of multilevel analysis. The results showed that the ICC (1) of proactive behavior was 0.09, the ICC (1) of self-efficacy was 0.11, and the ICC (1) of career development prospect was 0.14, both greater than 0.059. Therefore, the variance between groups cannot be ignored, and there has a need for multilevel analysis.

Hypothesis 1 predicted that HCWS has a significant positive impact on employees’ proactive behavior. As shown in model 2 in Table 3, after controlling the variables of industry, enterprise scale, and enterprise years at the enterprise level, as well as the variables of gender, age, and education level at the employee level, HCWS has a significant positive impact on employees’ proactive behavior (β = 0.086, *p* < 0.05), and Hypothesis 1 was supported.

**TABLE 3:** Regression results for testing hypotheses.

Variable	Proactive behavior									Career development prospect		Self-efficacy	
	Model 1	Model 2	Model 3	Model 4	Model 5	Model 6	Model 7	Model 8	Model 9	Model 10	Model 11	Model 12	Model 13
Intercept	5.649***	5.669***	2.382***	3.412***	2.065***	5.648***	5.647***	5.697***	5.728***	5.283***	5.311***	5.47***	5.493***
Organizational level													
Industry	–0.001	–0.011	0.009	0.014	0.018	0.004	0.004	0.001	–0.001	–0.022	–0.036	–0.047	–0.056
Enterprise scale	0.017	0.021	0.026	0.029	0.029	0.017	0.017	0.017	0.018	–0.023	–0.018	–0.008	–0.004
Enterprise years	−0.006*	−0.007*	–0.004	–0.004	–0.003	–0.005	–0.005	–0.005	–0.005	–0.007	−0.008*	–0.004	–0.005
High commitment work system		0.086*	0.040	0.040	0.026						0.114*		0.081*
Self-efficacy			0.601***		0.411***								
Career development prospect				0.422***	0.256***								
Individual level													
Gender	–0.023	–0.019	0.015	0.055	0.039	–0.011	–0.011	–0.027	–0.031	−0.195*	−0.190*	–0.066	–0.063
Age	0.010	0.010	–0.003	0.007	–0.001	–0.003	–0.003	–0.010	–0.011	0.003	0.003	0.016	0.016
Education level	–0.008	–0.013	–0.013	–0.013	–0.011	0.000	0.000	–0.001	–0.002	0.027	0.018	0.002	–0.003
Self-efficacy			0.672***		0.596***			0.577***	0.593***				
Career development prospect				0.340***	0.132***	0.259***	0.259***						
Comply with personality						0.407***	0.408***	0.221***	0.192***				
Self-efficacy × personality									−0.057*				
Career prospects × personality							0.003						
Total pseudor2	0.010	0.017	0.416	0.224	0.449	0.248	0.248	0.364	0.366	0.017	0.026	0.020	0.027
Changes of pseudor2	0.010	0.007	0.407	0.214	0.349	0.238	0.238	0.354	0.356	0.017	0.009	0.020	0.008

Level 1 is the individual level, n = 1,055; level 2 is the organizational level, n = 117. *p < 0.05, *p < 0.01, *p < 0.001.

Hypothesis 2 predicted the mediating role of self-efficacy between HCWS and proactive behavior. As shown in model 13 in Table 3, HCWS has a significant positive impact on employees’ self-efficacy (β = 0.081, *p* < 0.05). In addition, on the basis of model 2, when HCWS and self-efficacy (intragroup and intergroup) are added into the model (model 3), the relationship between HCWS and proactive behavior becomes insignificant (β = 0.040, N.S.), which indicates that self-efficacy plays a mediating role between HCWS and proactive behavior. Hypothesis 2 is supported. At the same time, the confidence interval of indirect effect estimated by the Monte Carlo method is [0.003, 0.098], excluding 0. Hypothesis 2 is supported.

Hypothesis 3 predicted the mediating role of career development prospect between HCWS and proactive behavior. As shown in model 11 in Table 3, HCWS has a significant positive correlation with career development prospect (β = 0.114, *p* < 0.05). In addition, when HCWS and career development prospect (intragroup and intergroup) were added to the model (model 4), the effect of HCWS on proactive behavior was no longer significant (β = 0.040, N.S.). Within the group, career development prospect have a significant positive impact on proactive behavior (β = 0.340, *p* < 0.001). Among the groups, career development prospect also has a significant positive impact on proactive behavior (β = 0.422, *p* < 0.001). Therefore, career development prospect plays a mediating role between HCWS and proactive behavior. Hypothesis 3 is supported. At the same time, the confidence interval of indirect effect estimated by the Monte Carlo method is [0.047, 0.095], excluding 0. Hypothesis 3 is supported again.

Hypothesis 4 predicted the negative moderating effect of conformity values between self-efficacy and proactive behavior. In this study, self-efficacy and conformity values are centralized, and the interaction items between them are formed. Then, the interaction items of self-efficacy and conformity values are added in turn. Based on the results shown in model 9 in Table 3, the interaction between self-efficacy and conformity values was significant (β = –0.057, *p* < 0.05). This shows that conformity values play a negative moderating role between self-efficacy and proactive behavior. Hypothesis 4 is supported. Similarly, Hypothesis 5 predicted the negative moderating effect of conformity value between career development prospect and proactive behavior. In this study, the interaction items of career development prospect and conformity values were added in turn. Based on the results shown in model 7 in Table 3, the interaction between career development prospect and conformity values was not significant (β = 0.003, N.S.). This shows that the conformity value does not play a moderating role between career development prospect and proactive behavior, and Hypothesis 5 is not supported.

**FIGURE 2:**
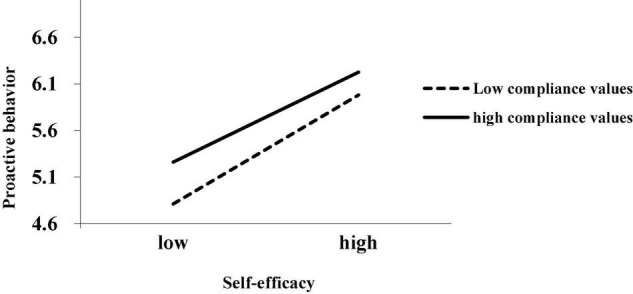
Interaction effect of self-efficacy and proactive behavior on conformity value.

To intuitively reflect the moderating effect of conformity value on self-efficacy and proactive behavior, this study drew a moderating effect diagram based on a positive and negative standard deviation of conformity value. As shown in Figure 2, when employees’ conformity values are low, the positive relationship between self-efficacy and proactive behavior is stronger. On the contrary, when employees’ conformity values are higher, the positive relationship between self-efficacy and proactive behavior is weaker. This further supports Hypothesis 4.

## Discussion

In this article, we explored the multilevel analysis method to explore the mechanism of HCWS on employees’ proactive behavior and clarify the complex influence of organizational factors and individual factors on proactive behavior.

### Theoretical Contribution

First, most of the existing studies on proactive behavior only focus on the influence of a certain kind of factors (e.g., organizational context or individual factors). Although the results of previous studies, organizational contextual factors (e.g., leadership style and job characteristics), and individual factors (e.g., gender, age, knowledge, and skills) can affect proactive behavior, there are relatively few studies on the complex influence of organizational factors and individual factors. In contrast, some scholars suggested that a strong situation can offset the influence of individual differences, while a weak situation can promote individual differences. For example, Grant and Sumanth (2009) found that individual tendency can complement a weak situation. When the trust tendency of individuals is high, even if their leaders are not credible, they will show a high level of work proactive. In contrast, some scholars point out that work situation factors can positively cooperate with individual factors to influence proactive behavior. For example, Griffin et al. (2010) found that leadership vision and employees’ high level of role breadth self-efficacy can significantly affect proactive behavior. Therefore, we should investigate the complex influence of organizational context factors and individual factors on proactive behavior more comprehensively. By integrating the organizational factor (e.g., HCWS) and the individual factor (e.g., conformity value), this study further examines the influence mechanism on proactive behavior, which will provide a new idea for organizational behavior research.

Second, most of the previous studies on proactive behavior focused on general cognitive motivation. However, these studies lacked the identification of specific cognitive motivation based on previous dependent variables. This study attempts to identify and verify the dual-mediating mechanism between HCWS and employee proactive behavior, which are typical cognitive motivation (self-efficacy) and special cognitive motivation (employees perceived career development prospect). As Batistič et al. (2016) pointed out, HRM system and relationship climate can interact with employee proactive behavior. This study comprehensively and systematically clarifies the relationship between HCWS (HCWS) and employees’ proactive behavior. Compared with HPWS and HIWS, HCWS emphasizes on building emotional relationship between organizations and employees. This study comprehensively examines the mechanism of HCWS on proactive behavior and identifies the dual-mediating mechanisms about self-efficacy and career development prospect.

Finally, previous studies on proactive behavior focused more on individual factors, such as individual statistical characteristics and personality, and paid less attention to individual values. However, individual values are the key factors that affect individual attitude and behavior (Schwartz, 1994). Different values lead to great differences in the process of individual goal and behavior. Even in the same organizational context, values as an individual judgment of “worth doing” will impact on the individual willingness to act deeply. The results show that the positive relationship between self-efficacy and proactive behavior is negatively regulated by individual values, while the positive relationship between career development prospect and proactive behavior is not negatively regulated by individual values. This not only widens the boundary conditions of proactive behavior research but also complements the existing research on the influence of individual factors on proactive behavior.

### Practical Implications

In practice, this study has brought a lot of enlightenment for enterprise managers.

1. To build a useful organizational context, this study can improve the motivation of “can do” and “have reason to do” of employees, so that they can be more involved in proactive behavior. In contrast, the organization can adopt an HCWS, implement HRM policies and measures oriented by employee commitment, and increase the joint connection between the organization and employees through a variety of ways, so that employees are willing to face the problems in their work more proactively for the sake of individual development and organizational progress. In contrast, the organization can take diversified measures to encourage employees to improve their knowledge and skills, such as supporting more information sharing among teams or groups, which will enhance their sense of self-efficacy and lay a solid foundation for better work in the future.
2. Enterprises need to consider the influence of individual factors while paying attention to the proactive behavior of employees. Combined with the individual value tendency of employees, it is suggested that enterprise managers should carefully examine those employees who have a high conformity value tendency. At the same time, we should create a safe and inclusive organizational culture to encourage employees to play their own proactive. Under the guidance of this culture, we believe that all employees in the organization are more willing to proactively face the daily work in order to pursue the win-win goal of their own development and organizational development.
3. We should respect the development and growth needs of employees and take measures to increase the consistency of organizational goals and individual goals. On the premise of not harming the interests of the organization, we should provide more growth platforms and opportunities for employees, which will increase the stickiness of existing employees to the organization, attract more potential new employees, and encourage all employees in the enterprise to work hard for the benefit of the organization.

### Limitations and Future Research

Although this study adopts a multisource and paired study design, there are still some limitations. First of all, in Hypotheses 4 and 5, this study proposes the negative moderating effect of conformity value on the relationship between self-efficacy and proactive behavior, career development prospect, and proactive behavior. The data analysis results show that Hypothesis 4 is supported, and conformity value can negatively regulate the positive relationship between self-efficacy and proactive behavior. However, Hypothesis 5 is not supported, and conformity value does not negatively regulate the positive relationship between career development prospect and proactive behavior. This may be due to the following reasons. First, self-efficacy represents the cognitive motivation of “being able to do,” and career development prospect represents the cognitive motivation of “having a reason to do.” Although both of them have a significant impact on proactive behavior, the cognitive motivation of “having a reason to do” is more important and has a more direct effect on proactive behavior. When the individual has the motivation of “having a reason to do,” in order to achieve the internal goal of the individual, less consideration is given to the uncertainty and possible problems. Therefore, the value judgment of whether it is “worth doing” may not have an impact. Second, this study only selects the variable “career development prospect” and does not examine other reasonable cognitive motivation. It is suggested that future research can examine other variables to verify the results of this study, such as psychological security, supervisor support, stress perception, and job wellbeing. Second, because the data of this study are cross-sectional but not cross-temporal, this study cannot verify the causality. Future studies can use longitudinal research design to further verify the causality between variables. Finally, although this study adds individual values into the overall framework, it does not explore the influence of other individual factors. Future research can continue to explore the complex influence of organizational context factors and individual factors on proactive behavior, so as to understand the process and mechanism of proactive behavior in more detail.

## Data Availability Statement

The raw data supporting the conclusions of this article will be made available by the authors, without undue reservation.

## Author Contributions

YS put forward the conceptual model and wrote the manuscript. MC collected the data and made valuable suggestions for both the initial draft and subsequent revisions. Both authors contributed to the article and approved the submitted version.

## Conflict of Interest

The authors declare that the research was conducted in the absence of any commercial or financial relationships that could be construed as a potential conflict of interest.

## Publisher’s Note

All claims expressed in this article are solely those of the authors and do not necessarily represent those of their affiliated organizations, or those of the publisher, the editors and the reviewers. Any product that may be evaluated in this article, or claim that may be made by its manufacturer, is not guaranteed or endorsed by the publisher.
